# Supplementary material for: Competitive repopulation of an empty microglial niche yields functionally distinct subsets of microglia-like cells
Source: Nat Commun. 2018 Nov 19;9:4845. doi: 10.1038/s41467-018-07295-7 (PMC6242869; doi:10.1038/s41467-018-07295-7)
Supplement: Supplementary file 1 — Supplementary Information [file 41467_2018_7295_MOESM1_ESM.pdf]

Supplementary Information

Competitive repopulation of an empty microglial niche yields functionally distinct subsets of microglia-like cells

Lund et al

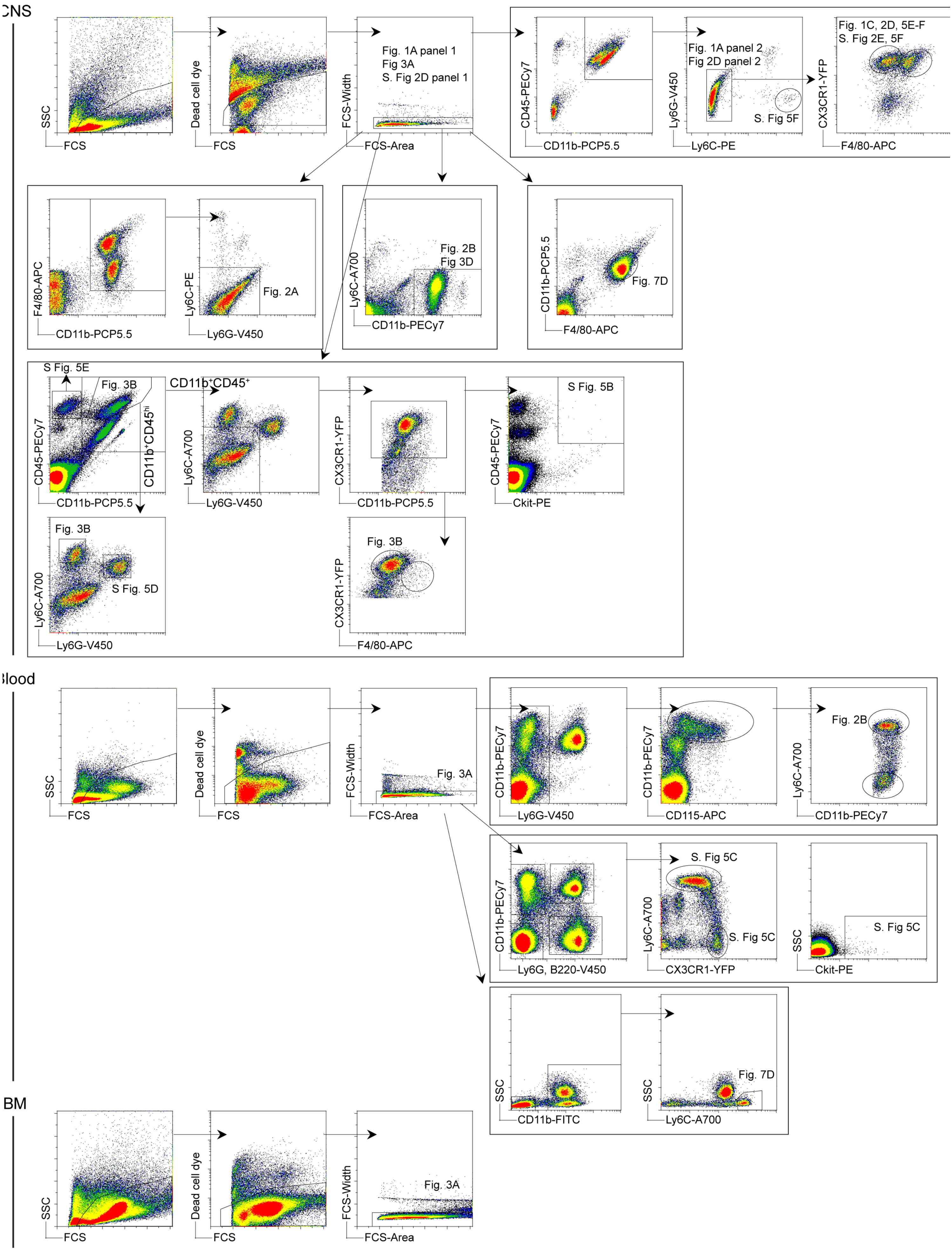

Supplementary Figure 1: Gating strategies
All gating strategies used in this study.

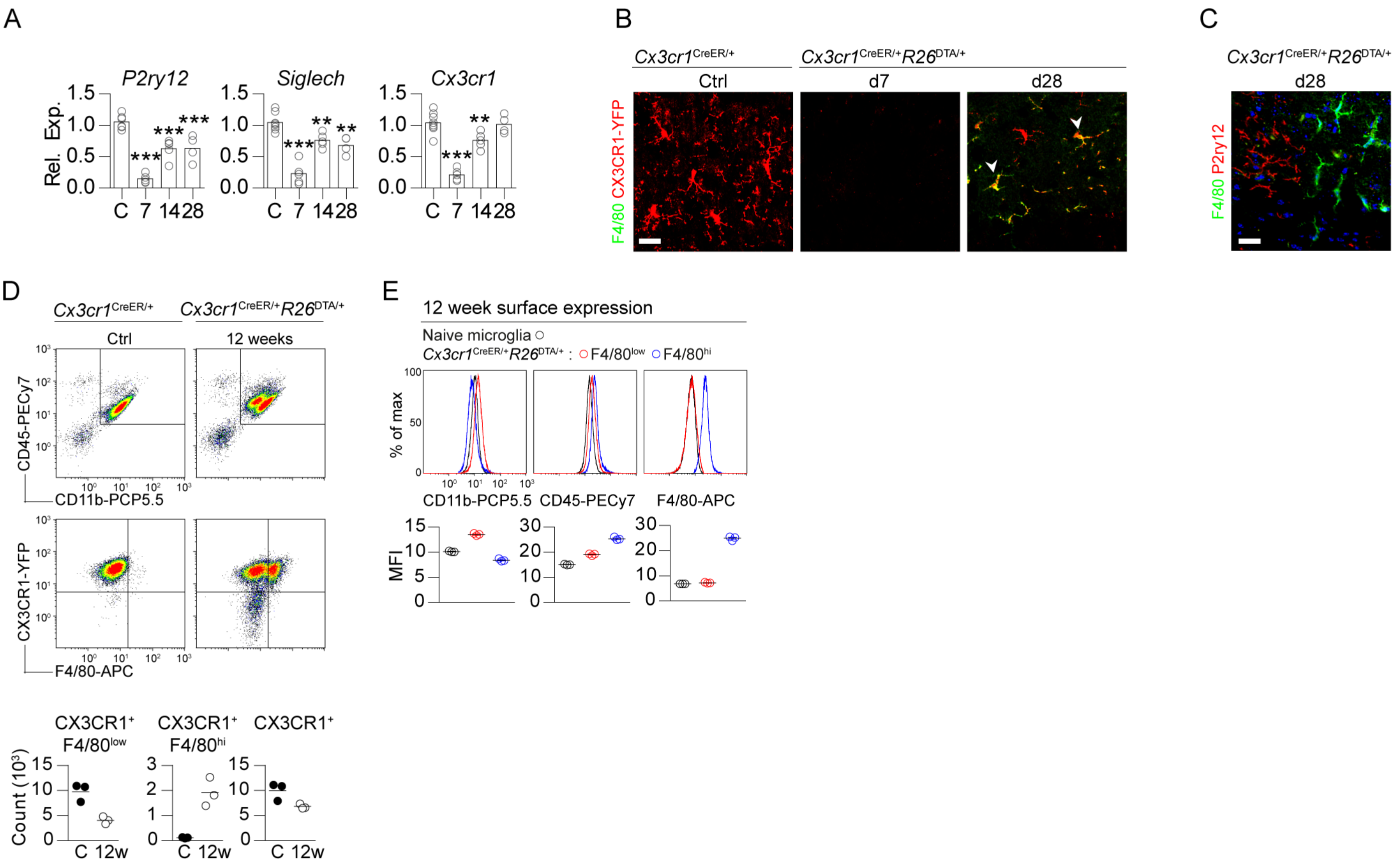

Supplementary Figure 2. Additional data related to Figure 1

(A) qPCR analysis of microglia-expressed genes in whole brain. n=8, 5, 5, 4 mice. Bars represent mean values. ***p<0.001 **p<0.01 by one-way ANOVA and Dunnett's Multiple Comparison test. (B) Immunofluorescence to CX3CR1-YFP and F4/80 in the hippocampus. The images are representative of n=3 mice/group. Scale bar 25µm. (C) Immunofluorescence of P2ry12 and F4/80 staining in the cortex at day 28. The images are representative of n=3 mice. Scale bar 25µm. (D) Analysis of CNS 12 weeks after TAM administration and quantification of $F4/80^{low}$ and $F4/80^{hi}$ subsets. Top panel is gated on live singlet cells. Bottom panel is gated on $CD11b^{+}CD45^{+}Ly6C^{-}Ly6G^{-}$. Lines represent mean values. The experiment was performed once. (E) Surface marker expression of $F4/80^{low}$ and $F4/80^{hi}$ 12 weeks after TAM administration. Lines represent mean values.

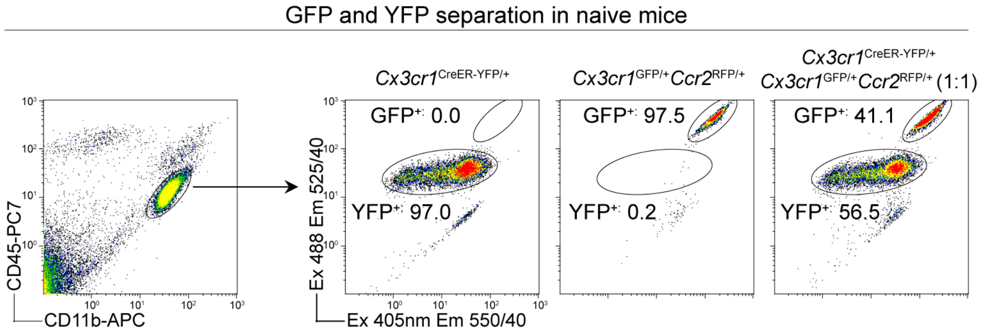

Supplementary Figure 3. Separation of GFP and YFP signals by flow cytometry
Successful separation of CX3CR1-GFP$^+$ and CX3CR1-YFP$^+$ microglia (CD11b$^+$CD45^{low}) by exciting GFP using the 405-nm violet laser and recording emission using the 550/40-nm filter. Related to experiments using chemotherapy (Fig. 2B) and adoptive transfer of monocytes (Fig. 3D).

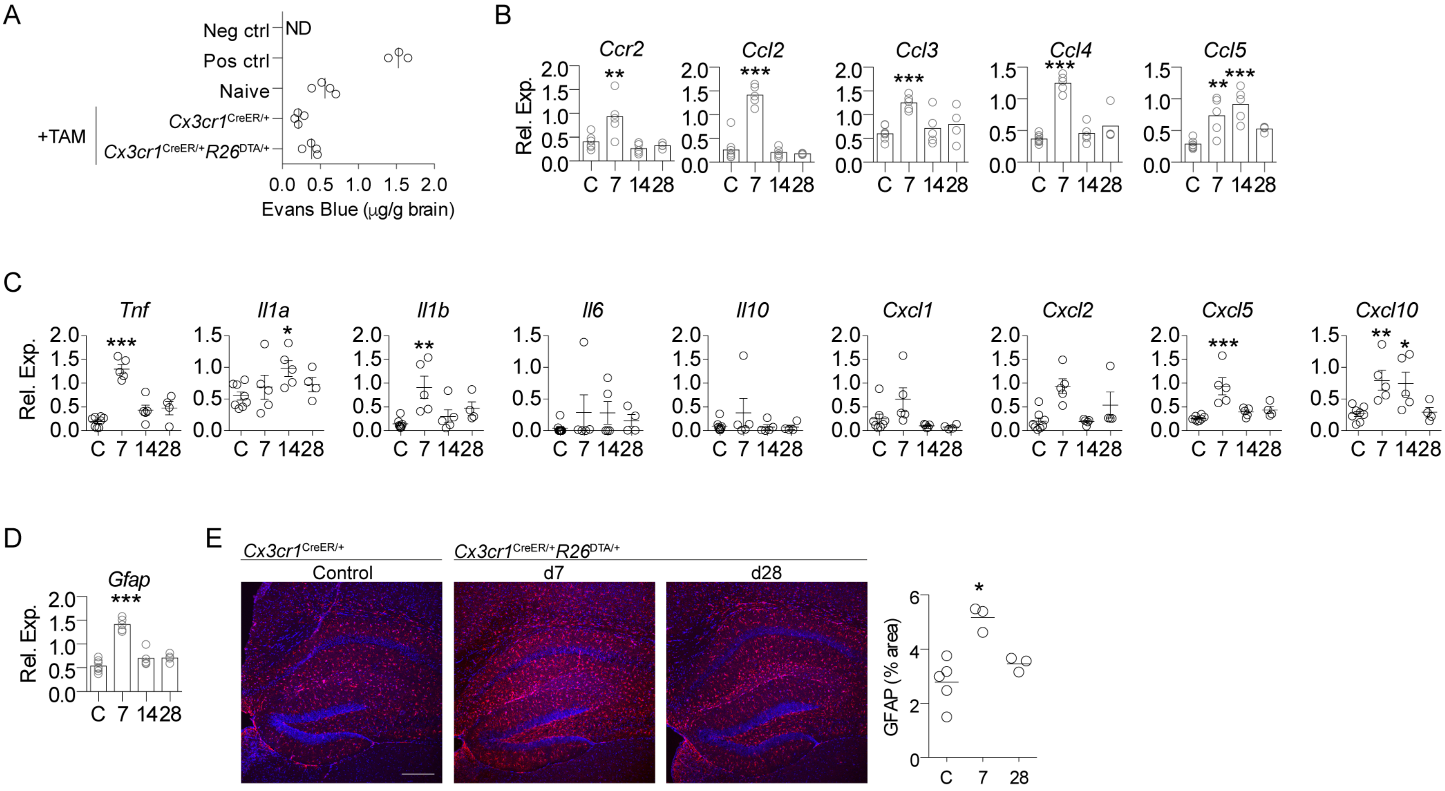

Supplementary Figure 4. BBB integrity and inflammatory changes in microglia depleted brains

(A) BBB integrity measured by the amount of Evans blue extravasation into the brain after 24 hours. Evans blue was injected into control *Cx3cr1*$^{\mathrm{CreER/+}}$ and *Cx3cr1*$^{\mathrm{CreER/+}}$*R26*$^{\mathrm{DTA/+}}$ mice 7 days after TAM administration. Naive mice did not receive TAM. Negative control mice were not injected with Evans blue. Positive control mice received Evans blue but were not perfused. n=3-4 mice/group. The experiment was performed once. Lines represent mean values. ND = not detectable. (B) qPCR analysis of *Ccr2* and monocyte chemoattractants *Ccl2, Ccl3, Ccl4 and Ccl5* in whole brain at the indicated time points after TAM. n= 8, 5, 5, 4 mice. Bars represent mean values. ***p<0.001 **p<0.01 by one-way ANOVA and Dunnett's Multiple Comparison test. (C) qPCR analysis of several inflammatory cytokines and chemokines. mean±s.e.m. ***p<0.001 **p<0.01 *p<0.05 by one-way ANOVA and Dunnett's Multiple Comparison test. (D) qPCR analysis of *Gfap*. Bars represent mean values. ***p<0.001 by one-way ANOVA and Dunnett's Multiple Comparison test. (E) Quantification of GFAP staining intensity in the hippocampus at day 7 and 28 after TAM administration. n=5, 3, 3 mice. Lines represent mean values. *p<0.05 by one-way ANOVA and Dunnett's Multiple Comparison test. Scale bar 200 µm.

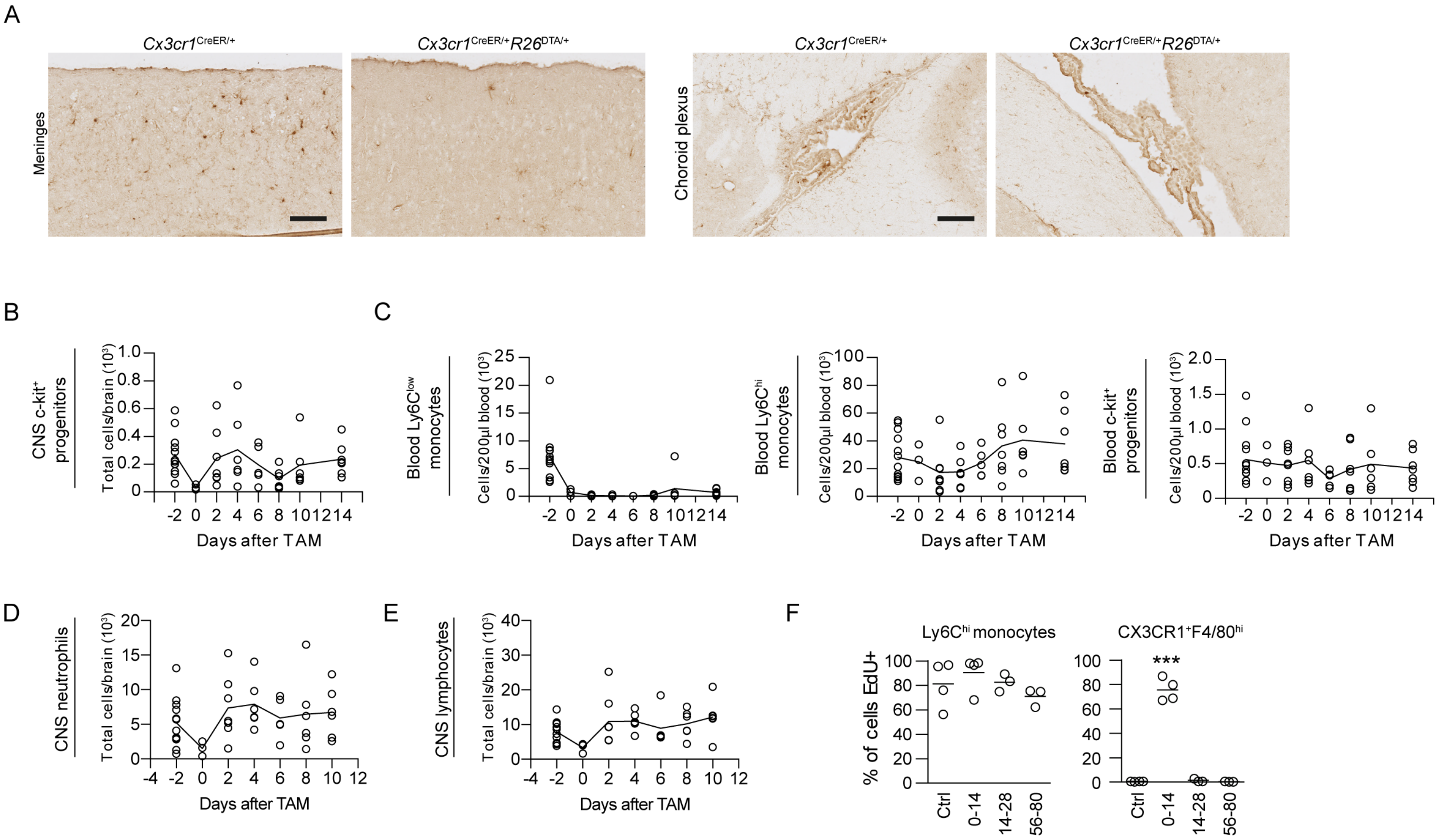

Supplementary Figure 5. Kinetic analysis of CNS and blood following depletion. (A) Iba-1 staining in meninges and choroid plexus on day 2 after TAM. Scale bar 100 µm (B-E) Analysis of CNS c-kit^{+} cells (B), blood Ly6C^{low} and Ly6C^{hi} monocytes and blood lin^{-}c-kit^{+} progenitors (C), CNS neutrophils (D) and CNS lymphocytes (E) during depletion. Gating strategies are detailed in Supplementary Fig. 1. Lines represent mean values. (F) Analysis of EdU incorporation in CNS retrieved Ly6C^{hi} monocytes and monocyte-derived F4/80^{hi} macrophages. EdU was administered in the drinking water for 14 days during the indicated time periods after TAM. Control mice were given EdU days 0-14 after TAM. Gated on CD11b^{+}Ly6C^{hi}Ly6G^{-} or CD11b^{+}Ly6C^{-}Ly6G^{-}CX3CR1^{+}F4/80^{hi}. n=3-4 mice/group, the experiment was performed twice. Lines represent mean values. *** $p<0.001$ by one-way ANOVA and Dunnett's Multiple Comparison test.

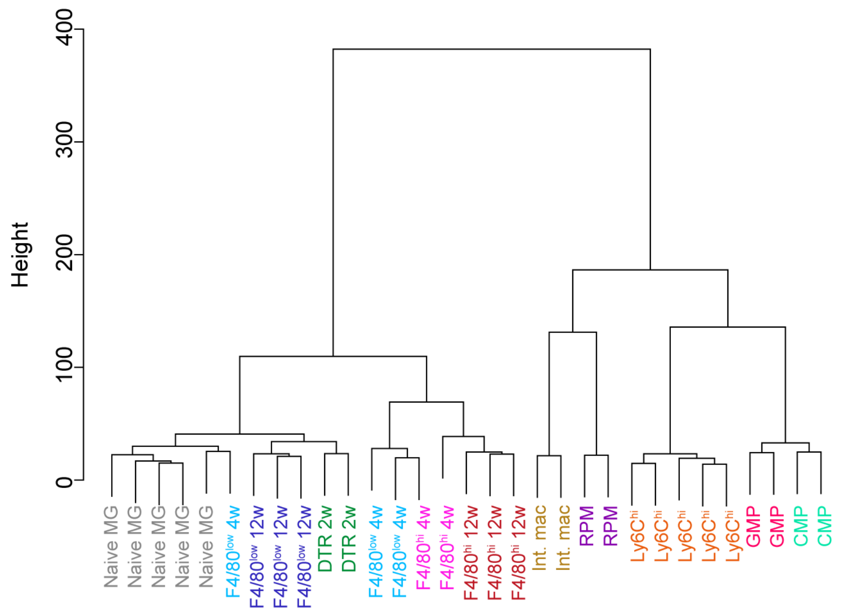

Supplementary Figure 6. Additional data related to Figure 4
Unsupervised hierarchical clustering of microarray expression profiles.

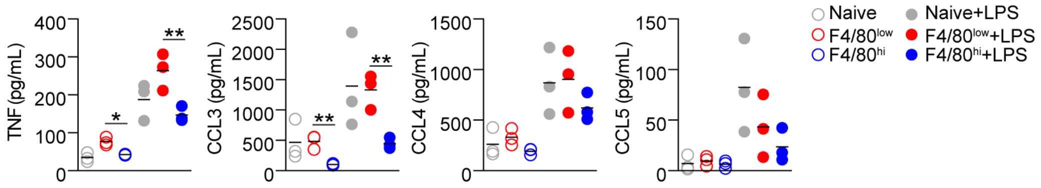

Supplementary Figure 7. Cytokine production by F4/80^{low} and F4/80^{hi} cells
Cytokine production measured using CBA in supernatants from *ex vivo* isolated microglia cultured overnight ± 100ng/ml LPS. Microglia were sorted from *Cx3cr1*$^{CreER/+}$ mice (naive) or *Cx3cr1*$^{CreER/+}$*R26*$^{DTA/+}$ (F4/80^{low} and F4/80^{hi}) 5 weeks after TAM. n=3mice/group. Lines represent mean values. *$p<0.05$ **$p<0.01$ by Student's two-tailed unpaired t-test.

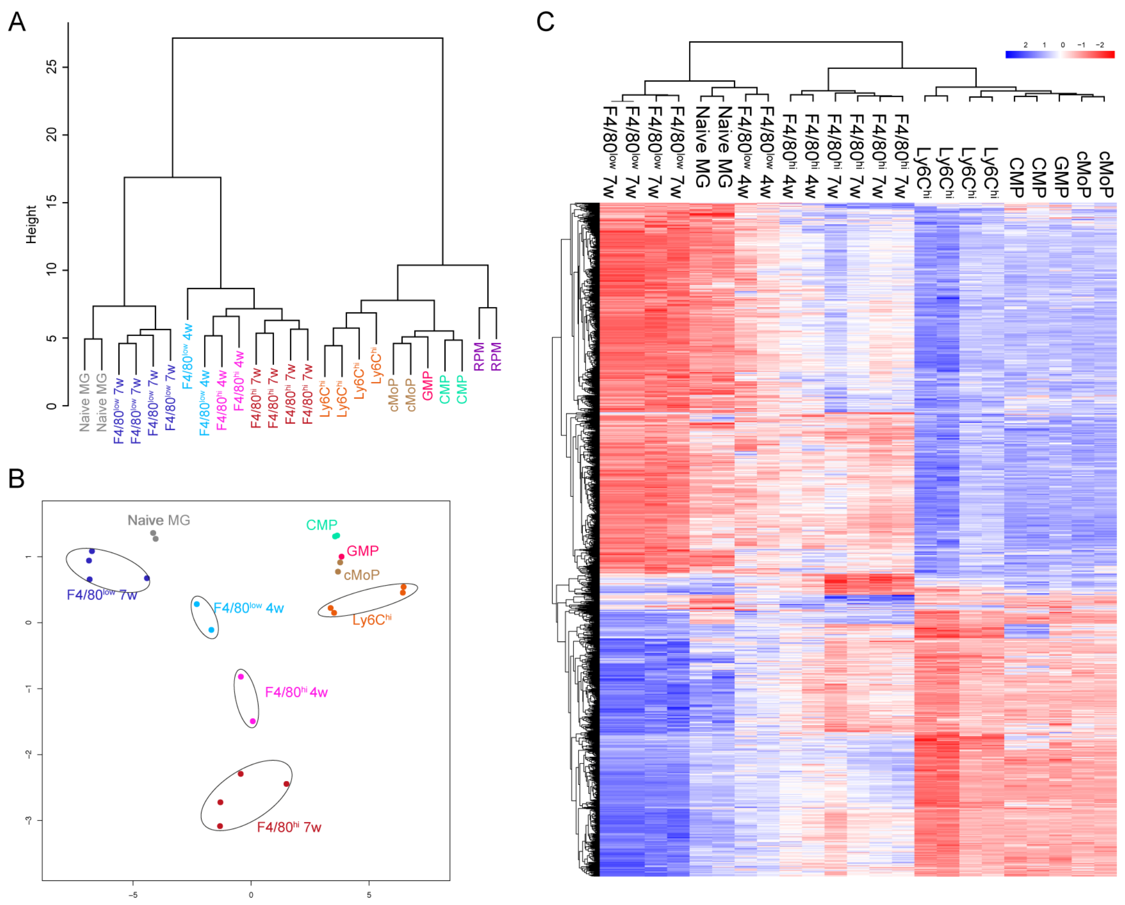

Supplementary Figure 8. DNA methylation profile of repopulating microglia/macrophages

(A) Unsupervised hierarchical clustering of DNA methylation profiles in sorted populations based on 17.614 CpG sites that passed QC. RPM, CMP, GMP, cMoP, Ly6C^{hi} monocytes and naive microglia (MG) were sorted from naive mice. cMoP = common monocyte progenitor. Each sample represents pools of 2-5 mice. (B) Multidimensional scaling (MDS) plot visualizing the 1000 most variable CpG sites in the dataset. (C) Heat map (row z-scores) of 1486 CpG sites identified as significantly differentially methylated (adj. p<0.001) between the three groups Ly6C^{hi} monocytes, F4/80^{low} microglia, F4/80^{hi} macrophages.

Supplementary Table 1

Competitive repopulation of an empty microglial niche yields functionally distinct subsets of microglia-like cells

Lund et al

**Gene**	**Forward Primer**	**Reverse Primer**
Ccl2	TTAAAAACCTGGATCGGAACCAA	GCATTAGCTTCAGATTTACGGGT
Ccl3	TTCTCTGTACCATGACACTCTGC	CGTGGAATCTTCCGGCTGTAG
Ccl4	TTCCTGCTGTTTCTCTTACACCT	CTGTCTGCCTCTTTTGGTCAG
Ccl5	TGCTGCTTTGCCTACCTCTC	TCCTTCGAGTGACAAACACGA
Ccr2	ATCCACGGCATACTATCAACATC	CAAGGCTCACCATCATCGTAG
Cx3cr1	GAGTATGACGATTCTGCTGAGG	CAGACCGAACGTGAAGACGAG
Cxcl1	CTGGGATTCACCTCAAGAACATC	CAGGGTCAAGGCAAGCCTC
Cxcl10	CCAAGTGCTGCCGTCATTTTC	GGCTCGCAGGGATGATTTCAA
Cxcl2	ACAGAAGTCATAGCCACTCTCA	TCAGACAGCGAGGCACATC
Cxcl5	TGCCCTACGGTGGAAGTCATA	TGCATTCCGCTTAGCTTTCTTT
Gapdh	TTCACCACCATGGAGAAGGC	GGCATGGACTGTGGTCATGA
Gfap	CCCTGGCTCGTGTGGATTT	GACCGATACCACTCCTCTGTC
Hprt	ACAGCCCCAAAATGGTTAAGG	TCTGGGGACGCAGCAACTGAC
Il10	GGTTGCCAAGCCTTATCGGA	ACCTGCTCCACTGCCTTGCT
Il1b	CTGTGTCTTTCCCGTGGACC	CAGCTCATATGGGTCCGACA
Il6	CCTCTCTGCAAGAGACTTCCATC	AGCCACTCCTTCTGTGACTCCAG
Ila	CAAACTGATGAAGCTCGTCA	TCTCCTTGAGCGCTCACGAA
P2ry12	CCCTGTGCGTCAGAGACTAC	CAAGCTGTTCGTGATGAGCC
Siglech	GGAGGCAAAACATGGAATTTCTG	CACATCACATTGGTAGGACGAC
Tnf	TACTGAACTTCGGGGTGATCGGTCC	CAGCCTTGTCCCTTGAAGAGAACC
