# Supplementary material for: Competitive repopulation of an empty microglial niche yields functionally distinct subsets of microglia-like cells
Source: Nat Commun. 2018 Nov 19;9:4845. doi: 10.1038/s41467-018-07295-7 (PMC6242869; doi:10.1038/s41467-018-07295-7)
Supplement: Supplementary file 8 — Reporting Summary [file 41467_2018_7295_MOESM8_ESM.pdf]

nature research

Corresponding author(s): Robert A. Harris

Reporting Summary

Nature Research wishes to improve the reproducibility of the work that we publish. This form provides structure for consistency and transparency in reporting. For further information on Nature Research policies, see Authors & Referees and the Editorial Policy Checklist.

Statistical parameters

When statistical analyses are reported, confirm that the following items are present in the relevant location (e.g. figure legend, table legend, main text, or Methods section).

n/a	Confirmed	
☐	☒	The exact sample size (n) for each experimental group/condition, given as a discrete number and unit of measurement
☐	☒	An indication of whether measurements were taken from distinct samples or whether the same sample was measured repeatedly
☐	☒	The statistical test(s) used AND whether they are one- or two-sided<br>*Only common tests should be described solely by name; describe more complex techniques in the Methods section.*
☒	☐	A description of all covariates tested
☐	☒	A description of any assumptions or corrections, such as tests of normality and adjustment for multiple comparisons
☐	☒	A full description of the statistics including central tendency (e.g. means) or other basic estimates (e.g. regression coefficient) AND variation (e.g. standard deviation) or associated estimates of uncertainty (e.g. confidence intervals)
☐	☒	For null hypothesis testing, the test statistic (e.g. F, t, r) with confidence intervals, effect sizes, degrees of freedom and P value noted<br>*Give P values as exact values whenever suitable.*
☒	☐	For Bayesian analysis, information on the choice of priors and Markov chain Monte Carlo settings
☐	☒	For hierarchical and complex designs, identification of the appropriate level for tests and full reporting of outcomes
☒	☐	Estimates of effect sizes (e.g. Cohen's d, Pearson's r), indicating how they were calculated
☐	☒	Clearly defined error bars<br>*State explicitly what error bars represent (e.g. SD, SE, CI)*

Our web collection on statistics for biologists may be useful.

Software and code

Policy information about availability of computer code

Data collection	Kaluza 1.3 (flow cytometry), BioRad CFXManager
Data analysis	Kaluza 1.3 (flow cyteometry), BioRad CFX Manager (qPCR), Partek Genomics Suite, Ingenuity Pathway Analysis, BubbleGum (Microarray), R using packages described in methods (DNA methylation), Pannoramic viewer and histoquant software (immunohistochemistry) GraphPad Prism 5.0 (statistics)

For manuscripts utilizing custom algorithms or software that are central to the research but not yet described in published literature, software must be made available to editors/reviewers upon request. We strongly encourage code deposition in a community repository (e.g. GitHub). See the Nature Research guidelines for submitting code & software for further information.

Data

Policy information about availability of data

All manuscripts must include a data availability statement. This statement should provide the following information, where applicable:

- Accession codes, unique identifiers, or web links for publicly available datasets
- A list of figures that have associated raw data
- A description of any restrictions on data availability

The data that support the findings of this study are available from the corresponding author upon reasonable request. Microarray and DNA methylation array raw

data have been deposited in the Gene Expression Omnibus data repository under accession number GSE121409 and GSE121483, respectively. A reporting summary for this article is available as a Supplementary Information file. The source data underlying figures 5D, 6A-E, 7A-B are provided as source data files

Field-specific reporting

Please select the best fit for your research. If you are not sure, read the appropriate sections before making your selection.

☒ Life sciences ☐ Behavioural & social sciences ☐ Ecological, evolutionary & environmental sciences

For a reference copy of the document with all sections, see nature.com/authors/policies/ReportingSummary-flat.pdf

Life sciences study design

All studies must disclose on these points even when the disclosure is negative.

Sample size	No sample size calculation was performed prior to experiments.
Data exclusions	in Fig 1D P2ry12 hippocampus, control group. (value=30.85). This sample was a significant outlier based on on GraphPad outlier calculator. This sample would actually increase the mean of the control group and we therefore thought it justifiable to exclude it. One sample was exculded from the microarray analysis because it was an outlier from all other samples. Several samples from the DNA methylation analysis were excluded due to chip failure.
Replication	All major experiments were repeated with similar results as stated in the figure legends.
Randomization	For each experiment mice were allocated into groups so that there would be close to equal representation of sex and age.
Blinding	Investigators were not blinded to group allocation

Reporting for specific materials, systems and methods

Materials & experimental systems

n/a	Involved in the study
☒	☐ Unique biological materials
☐	☒ Antibodies
☒	☐ Eukaryotic cell lines
☒	☐ Palaeontology
☐	☒ Animals and other organisms
☒	☐ Human research participants

Methods

n/a	Involved in the study
☒	☐ ChIP-seq
☐	☒ Flow cytometry
☒	☐ MRI-based neuroimaging

Antibodies

Antibodies used

Flow cytometry
B220-PercPCy5.5 (clone RA3-6B2, cat# 103236, dil 1/100, Biolegend)
B220-V450 (clone RA3-6B2, cat# 560473, dil 1/200, BD)
CD3-PercPCy5.5 (clone 17A2, cat# 100218, dil 1/100, BioLegend)
CD11b-PercPCy5.5, PE or PECy7 (clone M1/70, cat# 101228, 101208 or 101216 100, dil 1/100, BioLegend)
CD11c-A700 (clone N418, cat# 117320, dil 1/100, Biolegend)
CD16/32-PercPCy5.5 (clone 93, cat# 101324, dil 1/100, Biolegend)
CD34-eFluor660 (clone RAM34, cat# 50-0341-82, dil 1/100, Ebioscience)
CD36-A647 (clone MF3, cat# MCA2748A647, dil 1/100, Bio-Rad),
CD45-PECy7 or APCCy7 (clone 30F11, cat# 103114 or 103116, dil 1/100 BioLegend)
CD45.1-PEDazzle594 or APC/Cy7 (clone A20, cat# 110748 or 110716, dil 1/100 BioLegend)
CD45.2-PECy7 (clone 104, cat# 109830, dil 1/100, BioLegend)
CD115-APC (clone AFS98, cat# 135510, dil 1/100, BioLegend)
CD206-A647 (clone MR5D3, cat# 565250, dil 1/100, BD)
Ckit-PE (clone ACK2, cat# 135106, dil 1/100, Biolegend),
Clec12a-PE (clone 5D3/CLEC12A, cat# 143404, dil 1/100, Biolegend),
CXCR4-BV421, (clone 2B11, cat# 562738, dil 1/100, BD)
F4/80-PECy7 or APC (clone BM8, cat# 123114 or 123116, dil 1/100, Biolegend)
Ly6C-PE (clone HK1.4, cat# 128008, dil 1/250, BioLegend)
Ly6C-A700 (clone Al-21, cat# 561237, dil 1/100, BD Biosciences)

Ly-6G-V450 (clone 1A8, cat# 560603, dil 1/200, BD Biosciences)
MHCII-A700 (clone M5/114.15.2, cat# 107622, dil 1/100, Biolegend)
NK1.1-PCP5.5 (clone PK136, cat# 551114, dil 1/100, BD Biosciences).
Sca-1-PC7 (clone D7, cat# 108114, dil 1/100, Biolegend)
SiglecH-PE (clone 551, cat# 129606, dil 1/100, Biolegend)
TER119-PCP5.5 (clone TER-119, cat# 116228, dil 1/100, Biolegend).

Immunohistochemistry
Iba-1 (cat# 019-19741, dil 1/500, Wako)
F4/80-biotin (clone CI:A3-1, cat# MCA497B, dil 1/20, AbD Serotec)
P2ry12 (generated by O. Butovsky, dil 1/500)
GFP (cat# ab13970, dil 1/2000, Abcam)
GFAP (cat# ab7260, dil 1/2000, Abcam)
Ki67 (cat# ab15580, dil 1/200, Abcam)
ICAM-1 (clone YN1/1.7.4, cat# ab119871, dil 1/200, Abcam).

Validation

All antibodies used were validated in the literature or by suppliers.

Animals and other organisms

Policy information about studies involving animals; ARRIVE guidelines recommended for reporting animal research

Laboratory animals

All mice used were on the C57/B6 background. Both male and female mice were used and all experiments were started when mice were 6-12 weeks old. The following Jax-strains were used:
CX3CR1CreER: 021160
CX3CR1-GFP: 005582
R26DTR: 007900
R26DTA: 010527
CD45.1: 002014
CCR2-/-: 017586
Ifnar1-/- mice were originally obtained from Ulrich Kalinke

Wild animals

The study did not involve wild animals.

Field-collected samples

The study did not involve field-collected samples.

Flow Cytometry

Plots

Confirm that:

☒ The axis labels state the marker and fluorochrome used (e.g. CD4-FITC).

☒ The axis scales are clearly visible. Include numbers along axes only for bottom left plot of group (a 'group' is an analysis of identical markers).

☒ All plots are contour plots with outliers or pseudocolor plots.

☒ A numerical value for number of cells or percentage (with statistics) is provided.

Methodology

Sample preparation

This information is available under "preparation of single cell suspensions"

Instrument

Gallios (Beckman coulter) or Influx (BD)

Software

Kaluza 1.3 software (Beckman Coulter)

Cell population abundance

Post-sort purity >95% as described in methods.

Gating strategy

All data were first gated according to: FSC/SSC (remove debris) -> Dead cell/FCS (remove dead cells)-> FCS Width/FCS Area (remove dublets). The following gating strategies are provided in individual figures.

☒ Tick this box to confirm that a figure exemplifying the gating strategy is provided in the Supplementary Information.
